# A Quantitative Assessment of Tremor and Ataxia in Female *FMR1* Premutation Carriers Using CATSYS

**DOI:** 10.1155/2011/484713

**Published:** 2011-05-08

**Authors:** Vivien Narcisa, Dalila Aguilar, Danh V. Nguyen, Luis Campos, Jeffrey Brodovsky, Shana White, Patrick Adams, Flora Tassone, Paul J. Hagerman, Randi J. Hagerman

**Affiliations:** ^1^Medical Investigation of Neurodevelopmental Disorders (MIND) Institute, University of California-Davis Medical Center, 2825 50th Street, Sacramento, CA 95817, USA; ^2^Department of Pediatrics, University of California-Davis Medical Center, 2516 Stockton Boulevard, Sacramento, CA 95817, USA; ^3^Division of Biostatistics, Department of Public Health Sciences, School of Medicine, University of California-Davis, Davis, CA 95616, USA; ^4^Department of Biochemistry and Molecular Medicine, School of Medicine, University of California-Davis, Davis, CA 95616, USA

## Abstract

The fragile X-associated tremor/ataxia syndrome (FXTAS) is a relatively common cause of balance problems leading to gait disturbances in older males (40%) with the premutation. FXTAS is less common in females. We utilized the CATSYS system, a quantitative measure of movement, in 23 women with FXTAS (mean age 62.7; SD 12.3), 90 women with the premutation without FXTAS (mean age 52.9; SD 9.4), and 37 controls (mean age 56.53; SD 7.8). CATSYS distinguished differences between carriers with and without FXTAS in postural tremor, postural sway, hand coordination, and reaction time tasks. Differences were also seen between carriers without FXTAS and controls in finger tapping, reaction time, and one postural sway task. However, these differences did not persist after statistical correction for multiple comparisons. Notably, there were no differences across groups in intention tremor. This is likely due to the milder symptoms in females compared to males with FXTAS.

## 1. Introduction

Fragile X-associated tremor/ataxia syndrome (FXTAS) is caused by the premutation (55–200 CGG repeats) of the fragile X mental retardation 1 (*FMR1*) gene. FXTAS is more common in males than in females—among women with the premutation in families with known fragile X syndrome, 8–16% of women over age 50 develop FXTAS. Core symptoms of FXTAS include tremor and ataxia, although the syndrome also includes autonomic dysfunction, neuropathy, psychopathology, executive function deficits, and cognitive decline [1–3]. Individuals with FXTAS often present with balance problems that interfere with ambulation leading to frequent falls. The premutation expansion is associated with elevated *FMR1* mRNA that leads to a gain-of-function toxicity in neurons and astrocytes [4–8]. The RNA toxicity causes dysregulation of a number of proteins, including heat shock proteins (e.g., Hsp70), alpha B-crystallin, lamin A/C, and Sam68 and is associated with early cell death [9–11]. MRI findings include global brain atrophy and white matter disease in the middle cerebellar peduncles (MCPs), pons, periventricular area, subcortical regions, and insula, and on neuropathological assessment there are eosinophilic inclusions in neurons and astrocytes caused by the elevated levels of *FMR1*-mRNA [1, 12, 13]. 

Clinically noticeable tremor and ataxia typically begins in the early 60s, although some symptoms can be seen as early as the 40s [13]. Brain imaging studies, nerve conduction, psychiatric, and neuropsychological assessments suggest that there are early subclinical features of the premutation [3, 14–17]. A recent survey of 146 female carriers, compared to age-matched controls, demonstrated increased problems with balance, muscle pain, and intermittent tremor in carrier women who do not have FXTAS [18]. The current study suggests that a reliable quantitative measure of balance and tremor that is able to monitor early changes in the motor system could prompt early treatment. This is important because the core features of FXTAS are not as thoroughly described in the female premutation carrier population as in the male premutation carrier population. 

The CATSYS system is a set of computer-assisted diagnostic instruments that has been used for the quantitative evaluation of intention and postural tremor, postural sway, hand coordination, and reaction time [19]. The system is portable and can easily be transported to and set up in a variety of clinical settings. Administration of CATSYS is straightforward and, depending on the desired number of performance tasks, can be completed within a reasonable timeframe. This makes it an easy assessment for clinicians to quickly utilize and makes this system a simple yet comprehensive quantitative assessment tool for balance and movement. 

CATSYS has been utilized in two studies of male premutation carriers and identified a quantifiable difference between movements of male control patients and premutation patients with and without FXTAS [20, 21]. Allen et al. [21] found 70% concordance with CATSYS and self-report of ataxia and 80% concordance with CATSYS and self-report of tremor in carriers. Additionally, CATSYS detected ataxia in 30% of premutation men who did not self-report ataxia (*n* = 50) and 23% of premutation men who did not report tremor (*n* = 62), indicating that CATSYS not only detects FXTAS symptoms in men with FXTAS, but can be a useful tool in detecting preclinical motor symptoms of FXTAS. Aguilar et al. [20] also detected similar findings, especially in differences between men with FXTAS and controls with respect to intention tremor and postural sway area, and between men with FXTAS and carrier men without FXTAS with respect to postural sway. 

This study aims to investigate the potential use of the CATSYS system in female premutation carriers with and without evident clinical involvement of FXTAS. 

## 2. Materials and Methods

### 2.1. Subjects

Recruitment of patients occurred through the Fragile X Research Treatment Center at the MIND Institute of the University of California, Davis Medical Center (UCDMC). Subjects were recruited through family members of known probands with fragile X syndrome (FXS). Controls were recruited in a similar manner, or as wives of men with FXTAS.

After participants signed an informed consent, approved by the Institutional Review Board at the University of California, Davis Medical Center, a detailed medical history, neurological examination, and MRI when possible were used to diagnose the presence of FXTAS. Based on these assessments and carrier status, patients were identified as FXTAS, non-FXTAS carriers, or controls. In total, 150 females participated, 23 were premutation carriers with FXTAS (mean age 62.7 years; SD 12.3), 90 were premutation carriers without FXTAS (mean age 52.9 years; SD 9.4) and 37 were controls (mean age 56.5 years; SD 7.8).

### 2.2. Methods

The CATSYS protocol was established from its original publication, where the various tasks were grouped into the four existing categories of postural tremor, postural sway, manual coordination, and reaction time [22]. As previously described, the annexation of the fifth group, intention tremor, was necessary to better evaluate the dyskinesia observed in patients with FXTAS [20, 23]. 

The postural tremor, intention tremor, postural sway, manual coordination, and reaction time used in this study contain the same tasks that were used to assess tremor and ataxia in a study of male *FMR1* premutation carriers [20]. 

### 2.3. Molecular Analysis

DNA was isolated from peripheral blood leukocytes, where 5 mL of whole blood was processed using standard methods (Puregene and Purescripts Kits, Gentra Inc., Minneapolis, MN; Tempus Tubes, Applied Biosystems, Foster City, CA) [24]. For Southern blot analysis, 5–10 g of isolated DNA was digested with EcoR1 and Nru1. The probe used in the hybridization was the StB12.3 [24]. Details were previously described in [24]. Analysis and calculation of the repeat size were carried out using an Alpha Innotech FluorChem 8800 Image Detection System.

### 2.4. Statistical Analysis

The statistical analysis proceeds in three parts. The primary analysis focuses on group comparisons (FXTAS, non-FXTAS carriers, and controls) with respect to *a priori* selected CATSYS outcome measures that include postural and intention tremor intensity (in both dominant and nondominant hands) and postural sway area (30 and 60 sec, eyes open; 10 and 30 sec eyes closed). These *a priori* primary measures (listed in Table 2) were selected based on a previous study of CATSYS measures on premutation male carriers [20]. The primary analyses were based on an analysis of covariance (ANCOVA), which adjusts for age. There was complete overlap in age that allows for proper age adjustment in ANCOVA models. *P* values were adjusted for multiple testing using the Sidak stepdown method, which provides strong control of type I error. 

An exploratory secondary analysis compares study groups with respect to manual hand coordination, finger tapping, reaction time, and writing tremor in both dominant and nondominant hands (listed in Table 3). These were selected as secondary outcome measures due to previous report that did not find significant differences among FXTAS, non-FXTAS, and controls [20]. For two ambidextrous subjects, dominant and nondominant hand values were assigned the average of the left and right hand measurements. We note that for a few patients who had measurements that were extremely outlying (more than three times the interquartile range), analyses were repeated without the outliers. In all statistically significant results reported, the conclusions were not sensitive to the few outlying observations. 

We considered a third exploratory analysis to examine data reduction of additional CATSYS sway measures, jointly with variables in the primary and secondary analyses described above. These include mean sway and sway intensity, in addition to sway area. Thus, we used principal components analysis (PCA) to explain and partition observed variation in all aforementioned CATSYS tasks. To adjust for age potential effects, we apply the PCA to the partial correlation matrix, where the effect of age is removed. 

## 3. Results

### 3.1. Patient Characteristics

Patients were between the ages of 36 to 80 in the FXTAS (*N* = 23), non-FXTAS (*N* = 90), and control (*N* = 37) groups. Although the age range is the same for all groups, the mean age for FXTAS, non-FXTAS carriers, and control groups were 62.69 (SD 12.27), 52.89, (SD 9.40), and 56.53 (SD 7.79). Because differences in age among the groups were significant, the subsequent analysis reported below adjusted for age. As expected, there were no differences in *FMR1* expression, CGG, and activation ratio (AR) between premutation carriers with and without FXTAS.  Table 1 summarizes the study patient characteristics in more detail.

### 3.2. Primary Outcomes: Postural Sway Area, Intention, and Postural Hand Tremor

**Figure 1:**
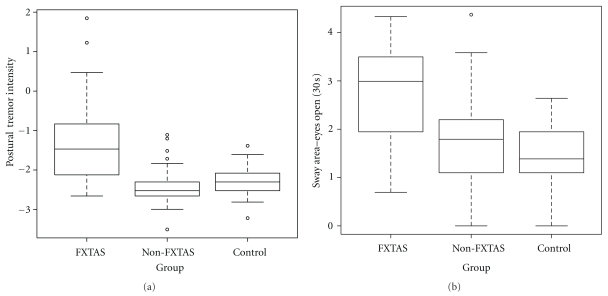
(a) Distribution/boxplots of (log) sway area (mm^2^) for eyes open 30 sec. task. Sway area was significantly higher in patients with FXTAS, compared to controls (*P* < .0001), as well as non-FXTAS (*P* < .0001). (Note: results unaffected by an outlying observation.) (Interior box horizontal bar = median). (b) Distribution/boxplots of (log) postural tremor intensity (m/sec^2^) in dominant hand (similar results for nondominant hand). Postural tremor intensity was significantly higher in patients with FXTAS, compared to controls (*P* < .0001), as well as non-FXTAS (*P* < .0001). (Note: results unaffected by outlying observations.) (Interior box horizontal bar = median).

Our primary outcomes are in the categories of sway area and hand tremor (intention and postural hand tremor intensity in dominant and nondominant hands). Postural hand tremor (intensity) was significantly greater in FXTAS, compared to controls (*P* < .0001) and non-FXTAS (*P* < .0001), although there was no difference observed between non-FXTAS and controls (see Table 2 and Figure 1(a)). These differences were observed in both dominant and nondominant hands. With respect to intention hand tremor, the data indicates that the levels among the three groups were not different.

Postural sway results showed similar, significantly higher sway area for patients with FXTAS compared to controls (*P* < .0001) or patients with non-FXTAS (*P* < .001) in all postural sway tasks (30 and 60 seconds eyes open; 10 and 30 seconds eyes closed). Table 2 and Figure 1(b) illustrate details of postural sway results. Note, for example in the 60 second eyes open task, that carriers with FXTAS, on average, have sway area about 4 times higher compared to non-FXTAS carriers. Typically postural sway areas are several folds higher for premutation carriers with FXTAS across all sway tasks compared to controls. Although not statistically significant after *P* value adjustment for multiple testing, there is a consistent trend that non-FXTAS carriers also showed higher postural sway (60 sec, *P* = .0756; 30 sec eyes closed, *P* = .0295) compared to controls.

### 3.3. Secondary Outcomes: Hand Coordination, Writing Tremor, and Reaction Time

Based on previous preliminary (nonsignificant) findings on a much smaller cohort of male premutation carriers [20], we defined *a priori* a set of secondary CATSYS outcome measurements of manual coordination (pronation-supination hand tapping and index finger tapping), writing tremor, and reaction time to an auditory stimulus (Table 3). With the larger sample size in the current study, there is a clear trend in differences in manual coordination (pronation-supination hand tapping and index finger tapping) and reaction time with significance (unadjusted *P* values, all *P* < .05) and with significantly decreased manual coordination as measured by finger tapping task in FXTAS compared to controls (*P* = .0024; significant after *P* value adjustment).

### 3.4. Exploratory Analysis of CATSYS Domains of Variation

**Figure 2:**
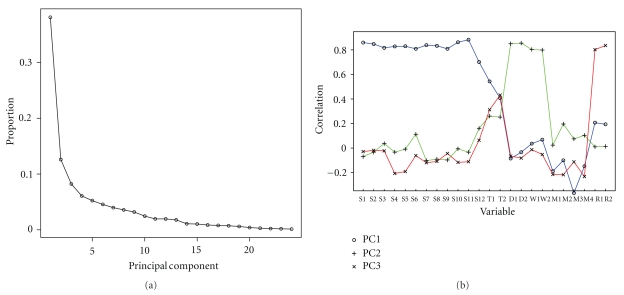
(a) Proportion of variation explained by each principal component based on all 24 CATSYS measures, and adjusted for age. The first 3 principal components account for the majority total data variation (*∼*60%). (b) The 24 individual variables of the CATSYS and their correlation to the first 3 principal components. The first PC (PC1) summarizes predominantly all sway tasks; the second PC (PC2) is primarily correlated with intention tremor (DotTrem) and writing tremor (WritTrem) in both hands; the third PC (PC3) captures association with postural tremor (TremInt) and reaction time in both hands. S1: SwayO60_Mean_, S2: SwayO60_Area_, S3: SwayO60_Int_, S4: SwayO30_Mean_, S5: SwayO30_Area_, S6: SwayO30_Int_, S7: SwayC30_Mean_, S8: SwayC30_Area_, S9: SwayC30_Int_, S10: SwayC10_Mean_, S11: SwayC10_Area_, S12: SwayC10_Int_, T1: TremInt_Dom_, T2: TremIntNon_Dom_, D1: DotTremInt_Dom_, D2: DotTremIntNon_Dom_, W1: WritTremInt_Dom_, W2: WritTremIntNon_Dom_, M1: Maxfreq_Dom_, M2: MaxfreqNon_Dom_, M3: MaxfreqTap_Dom_, M4: MaxfreqTapNon_Dom_, R1: ReactAvg_Dom_, R2: ReactAvgNon_Dom._

The CATSYS system provides numerous measures of sway on the force plate, including sway area, mean sway (defined as the average distance from the geometrical mean force center) and sway intensity (defined as the root mean square of the accelerations). In this exploratory analysis, we use principal components analysis (PCA, adjusted for age) to reduce the 24 CATSYS measures to a few summary variables (principal components), which includes all CATSYS measures in the primary and secondary analysis along with sway mean, intensity, and area. Briefly, PCA sequentially finds the optimal (linear) combinations of all input variables (i.e., the 24 CATSYS variables) where optimality is defined by maximization of variation. (These combinations are often also referred to as factor variables or simply as principal components.) Thus, the first principal component (PC) captures the most dominant (highest) sources of variation, and the second PC captures the second largest sources of variation (independent of the first), and so on. This can be seen in Figure 2, which displays the proportion of total variation explained by each principal component (PC) and shows that the first 3 PCs explain the majority of total variation (*∼*60%). (As defined by PCA, the first PC captures the largest variation, followed by the second PC, and the variation explained declines with each subsequent PC). To better understand the patterns of the 24 individual variables recorded by the CATSYS, one can examine each variable by comparing it to a principle component. Because each principle component represents either a large difference between numbers or a smaller difference between numbers, then each individual variable will closely match one over another. The resulting pattern in Figure 2(b) shows which CATSYS variables are most similar to each other by correlating with a peak in a particular principle component. The first PC summarizes predominantly all sway tasks, while the second PC primarily accounts for intention tremor and writing tremor in both hands. The third PC is primarily associated with postural tremor and reaction time in both hands. 

## 4. Discussion

Research regarding FXTAS in female premutation carriers remains a relatively small body of work because only 8 to 16% of older female carriers develop this problem [18, 25]. In this study, we have provided quantitative assessment of tremor and ataxia in a large cohort of female premutation carriers using the CATSYS system. Our findings demonstrate significant differences between those with FXTAS compared to controls, including postural sway, postural hand tremor intensity, and finger tapping. However, significant differences were not seen in the intention tremor measure. This is in contrast to previous reports of tremor in the males with FXTAS and perhaps this relates to the milder symptoms seen in female carriers [14, 20, 21, 26]. The milder symptoms seen in females with FXTAS compared to males with FXTAS are likely due to the protective effect of the normal X chromosome which diminishes the phenotype compared to males with FXTAS. The CNS changes in females are less severe than what is seen in males with FXTAS, including the degree of atrophy of the cerebellum and the severity of white matter disease [26]. Although we did not see a significant difference in the intention tremor rating across groups, we detected an array of motor findings that were more robust than previous studies in males with FXTAS on the CATSYS likely because of our larger cohort [20, 21]. 

There was limited evidence that females without FXTAS had differences in finger tapping, postural sway for 30 sec with eyes closed and reaction time, but these did not hold up with the statistical correction for multiple comparisons.

The secondary outcome measures, particularly the finger tapping and the reaction time, suggest subtle motor coordination involvement in carriers without FXTAS compared to controls. This is a particularly interesting finding and deserves further study including MRI measures, such as diffusion tensor imaging (DTI), to detect subtle abnormalities in white matter orientation that might represent a pre-FXTAS stage. In this regard, CATSYS may be useful in identifying pre-FXTAS individuals, giving movement clinics the ability to identify or screen for more individuals at risk for developing FXTAS. Furthermore, quantitative characterization of this subtle finding would be useful for designing early intervention studies with neuroprotective agents to hopefully alleviate the further progression of FXTAS [27]. These widespread motor problems captured by the CATSYS are supported by the broadened spectrum of neuropathological complications recently reported in females with and without FXTAS [18]. These symptoms include neuropathic pain and numbness suggestive of neuropathy, muscle pain, and hypertension. The broad clinical symptoms of FXTAS represent the RNA toxicity that occurs in a number of tissues besides the CNS [28]. The inclusions of FXTAS occur not only in neurons and astrocytes, but also in peripheral tissues including the thyroid, adrenal gland, testicles, and myenteric plexus of the gastrointestinal system (Greco et al. and Willemsen et al., personal communication) [28–30]. It is possible that thyroid disease, in addition to cochlear, eighth nerve, or inner ear problems associated with tinnitus, vertigo, and dizziness may also affect balance or even tremor, and such difficulties should be investigated in those with the premutation that, in our experience, commonly reports such symptoms.

We have found CATSYS to be a robust and quantitative measure of neurological dysfunction in premutation carriers, particularly those with FXTAS. We have also utilized this tool to follow patients with FXTAS who have been treated medically [27, 31]. In one case, we found improvement in FXTAS symptoms with venlafaxine and memantine treatment in follow-up studies after one year [31]. We suggest that this measure be utilized in the controlled treatment trials that are necessary to validate future treatments of FXTAS. 

##  CATSYS Manufacturing Information

For manufacturing supplies and details, please refer to the Danish Product Development Ltd. website at http://www.catsys.dk/purchase.htm. Budgeting for the complete system used in this investigation should be maximized at 10,000 Euros, or under 15,000 US dollars, based on current exchange rates.

##  Disclosure Statement

This project was conducted without any relationship between investigators and the CATSYS manufacturers and distributors. There was no discount or prior agreement to using the CATSYS for this investigation, nor are the investigators shareholders or consultants to the company.

## Figures and Tables

**Table 1:** Patient characteristics.

	Controls (*N* = 37)	Non-FXTAS (*N* = 90)	FXTAS (*N* = 23)	*P* value
Age	56.53 (7.79)	52.89 (9.40)	62.69 (12.27)	<.0001^a^
Education	14 (3.02)	15.35 (2.91)	14.66 (2.45)	.1692^a^
Ethnicity	(*N* ^c^ = 31)	(*N* = 87)	(*N* = 22)	
Caucasian	93.5% (29)	82.8% (72)	100% (22)	—
Hispanic	6.4% (2)	12.6% (11)	0%	—
Other	0%	4.9% (4)	0%	—
CGG Repeat	29.61 (5.65)	86.66 (17.01)	90.52 (31.38)	.3917^b^
*FMR1* mRNA	1.72 (0.75)	2.51 (0.94)	2.24 (0.84)	.2474^b^
AR		0.54 (0.18)	0.58 (0.21)	.3712^b^

^a^Overall ANOVA *P* value.

^b^Comparison between FXTAS and non-FXTAS.

^c^Less than total sample sizes due to missing ethnicity data.

**Table 2:** Comparison of *a priori* primary outcome CATSYS measures.

		Control (*N* = 37)		Non-FXTAS (*N* = 90)		FXTAS (*N* = 23)				
CATSYS group	CATSYS performance task	Mean	SD	Mean	SD	Mean	SD	FXTAS versus control	Non-FXTAS versus control	FXTAS versus non-FXTAS
Postural Tremor, Tremor intensity (m/sec^2^)	Hand:									
	Dominant	0.11	0.04	0.10	0.05	0.69	1.42	_∗∗∗_	ns^a^	_∗∗∗_
	Nondominant	0.10	0.04	0.09	0.04	0.98	2.51	_∗∗∗_	ns	_∗∗∗_

Intention Tremor, Tremor intensity (m/sec^2^)	Hand:									
	Dominant	2.24	1.47	2.14	1.31	2.33	2.50	ns	ns	ns
	Nondominant	2.09	1.07	2.20	1.10	2.54	2.21	ns	ns	ns

Postural sway area (mm^2^)	60 Sec:									
	Eyes open	7.94	4.29	11.21	9.99	41.95	35.04	_∗∗∗_	0.0756	_∗∗∗_
	30 Sec:									
	Eyes open	5.17	3.24	7.97	9.78	25.41	22.25	_∗∗∗_	ns	_∗∗∗_
	Eyes closed	8.86	6.57	14.42	13.33	48.32	46.59	_∗∗∗_	0.0295	_∗∗∗_
	10 Sec:									
	Eyes closed	5.77	3.61	11.00	16.27	46.81	55.49	_∗∗∗_	ns	_∗∗∗_

Analysis based on logarithm transformed data and adjusted for age.

^a^Not significant (*P* > .10).

***P < .0001 (significant after *P* value adjustment for multiple testing).

**Table 3:** Exploratory comparison of secondary outcome CATSYS measures.

		Control (*N* = 37)		Non-FXTAS (*N* = 90)		FXTAS (*N* = 23)				
CATSYS group	CATSYS performance task	Mean	SD	Mean	SD	Mean	SD	FXTAS versus control	Non-FXTAS versus control	FXTAS versus non-FXTAS
Manual coordination, Maximum frequency (Hz)	Hand									
	Dominant	7.07	0.82	6.82	0.98	6.42	1.14	_∗_	ns^a^	ns
	Nondominant	6.90	0.93	6.60	1.01	6.20	1.36	_∗_	ns	ns
	Finger tapping									
	Dominant	7.46	1.10	7.25	1.38	6.50	1.73	_∗_	_∗_	ns
	Nondominant	7.19	1.36	7.31	1.27	6.32	1.75	_∗_	_∗∗_	ns

Reaction Time (sec)	Hand									
	Dominant	0.24	0.05	0.24	0.05	0.27	0.05	_∗_	ns	ns
	Nondominant	0.23	0.05	0.24	0.05	0.26	0.05	_∗_	_∗_	ns

Writing Tremor, tremor Intensity (m/sec^2^)	Hand									
	Dominant	0.53	0.47	0.74	1.03	0.93	1.19	ns	ns	ns
	Nondominant	0.59	0.54	0.70	0.94	1.00	1.15	ns	ns	ns


Analysis based on logarithm transformed data and adjusted for age.

^a^ Not significant (*P* > .10).

**P < .01.

*P < .05.
